# Transposon-induced methylation of the *RsMYB1* promoter disturbs anthocyanin accumulation in red-fleshed radish

**DOI:** 10.1093/jxb/eraa010

**Published:** 2020-01-21

**Authors:** Qingbiao Wang, Yanping Wang, Honghe Sun, Liang Sun, Li Zhang

**Affiliations:** 1 National Engineering Research Center for Vegetables, Beijing Academy of Agriculture and Forestry Sciences, Beijing, China; 2 Key Laboratory of Biology and Genetic Improvement of Horticultural Crops (North China), Ministry of Agriculture, Beijing, China; 3 Beijing Key Laboratory of Vegetable Germplasm Improvement, Beijing, China; 4 Department of Vegetable Science, College of Horticulture, China Agricultural University, Beijing, China; 5 CONICET-National University of La Plata, Argentina

**Keywords:** CACTA transposon, DNA methylation, radish, (Raphanus sativus), *RsMYB1*, taproot flesh color

## Abstract

Red-fleshed radish (*Raphanus sativus* L.) is a unique cultivar whose taproot is rich in anthocyanins beneficial to human health. However, the frequent occurrence of white-fleshed mutants affects the purity of commercially produced radish and the underlying mechanism has puzzled breeders for many years. In this study, we combined quantitative trait location by genome resequencing and transcriptome analyses to identify a candidate gene (*RsMYB1*) responsible for anthocyanin accumulation in red-fleshed radish. However, no sequence variation was found in the coding and regulatory regions of the *RsMYB1* genes of red-fleshed (MTH01) and white-fleshed (JC01) lines, and a 7372 bp CACTA transposon in the *RsMYB1* promoter region occurred in both lines. A subsequent analysis suggested that the white-fleshed mutant was the result of altered DNA methylation in the *RsMYB1* promoter. This heritable epigenetic change was due to the hypermethylated CACTA transposon, which induced the spreading of DNA methylation to the promoter region of *RsMYB1*. Thus, *RsMYB1* expression was considerably down-regulated, which inhibited anthocyanin biosynthesis in the white-fleshed mutant. An examination of transgenic radish calli and the results of a virus-induced gene silencing experiment confirmed that *RsMYB1* is responsible for anthocyanin accumulation. Moreover, the mutant phenotype was partially eliminated by treatment with a demethylating agent. This study explains the molecular mechanism regulating the appearance of white-fleshed mutants of red-fleshed radish.

## Teaser

A CACTA transposon-induced methylation of the *RsMYB1* promoter might be associated with the production of white-fleshed mutants in red-fleshed radish.

## Introduction

Red-fleshed radish (*Raphanus sativus* L.), which has green or red skin and red flesh, is a unique cultivar grown in China, where it is consumed in large quantities because it is rich in anthocyanins. The main anthocyanin in red-fleshed radish is pelargonidin, with a content of approximately 4.69 mg g^–1^ fresh weight (Park *et al.*, 2011). In addition to their roles as plant pigments, anthocyanins are well known for their health benefits, such as antioxidant activity, anti-cancer effects, and enhancement of immunity (Butelli, 2008; He and Giusti, 2010).

The anthocyanin biosynthetic pathway has been elucidated in various plant species (Shi and Xie, 2014). Transcriptional regulation related to anthocyanin biosynthesis has also been extensively studied, with research indicating that the R2R3-MYB and bHLH transcription factors have a regulatory role as part of the MYB-bHLH-WD40 complex (Koes *et al.*, 2005; Petroni and Tonelli, 2011). Two genes, *Rc* (bHLH) and *Rd* (DFR), affecting proanthocyanidin synthesis in red and brown rice, have been analyzed. Specifically, previous studies showed that a 14 bp deletion in the *Rc* gene, which introduces a premature stop codon, alters the pericarp color from red to white (Sweeney, 2006; Furukawa *et al.*, 2007). In grape, insertion of a retrotransposon in the *VvMYBA1* promoter region and two non-conservative mutations in the adjacent *VvMYBA2* gene reportedly causes a change in skin color from red to white (Walker *et al.*, 2007). A 4 bp insertion in the *SlMYBATV* transcript, which encodes an R3 MYB repressor, leads to the up-regulated expression of anthocyanin biosynthesis genes in tomato (Cao *et al.*, 2017). In purple cauliflower, anthocyanin was observed to accumulate in response to the insertion of a Harbinger DNA transposon in the upstream regulatory region of a gene encoding a MYB transcription factor (Chiu *et al.*, 2010).

Epigenetic modifications of the genes encoding regulators of anthocyanin biosynthesis have been strongly linked to color changes in some crops. Altering the DNA methylation status of these regulatory genes affects the activities of the anthocyanin biosynthetic pathway. Methylation of the *PcMYB10* promoter is associated with green-skinned sports in a red pear cultivar (Wang *et al.*, 2013). Hypermethylation of the *MdMYB10* promoter results in a striped pigmentation of Honey Crisp apple fruit (Telias *et al.*, 2011). In maize, the *P1-wr* allele is hypermethylated compared with *P1-rr*, and diverse patterns of pigment accumulation are due to the epigenetic regulation of *p1* (Chopra *et al.*, 1998; Sidorenko *et al.*, 2000). A recent study determined that *p1* expression can be regulated by the DNA methylation status of the CACTA transposon inserted in the *Ufo1-1* gene, resulting in an unstable purple phenotype in maize (Wittmeyer *et al.*, 2018).

In radish, the *RsMYB1* and *RsTT8* genes have been cloned, and co-expression of these two genes in tobacco leaves has confirmed that they contribute to the regulation of anthocyanin biosynthesis (Lim *et al*., 2016, 2017). *RsMYB90/RsMYB1* is a crucial determinant of anthocyanin accumulation in red-skinned radish (Yi *et al.*, 2018; Luo *et al.*, 2020). Another anthocyanin biosynthesis gene, *RsF3′H*, was responsible for cyanidin-based anthocyanin contents in purple-fleshed radish (Masukawa *et al.*, 2018). Moreover, anthocyanin biosynthesis-related genes and microRNAs have been identified, and the associated coordinated regulation has been revealed by transcriptome analysis in red-fleshed or red-skinned radish (Muleke *et al.*, 2017; Sun *et al*., 2017, 2018; Liu *et al.*, 2019).

‘Xinlimei’ is a special red-fleshed radish landrace that originated in Beijing, whose taproot flesh colors vary (e.g. light green, red, and unevenly red) (BVRC and BAAFS, 1977). Systematic selection during breeding has resulted in the development of commercial red-fleshed varieties with uniformly red flesh and green skin. However, white-fleshed or red-skinned types appear occasionally in response to changes in the cultivation conditions. A 3-year analysis revealed a mutation rate of 0.49–0.57% for MTH01 (a red-fleshed inbred line that was self-crossed for more than 10 generations), producing mutants including red-skinned, white-fleshed, and chimeric types (see Supplementary Table S1 at *JXB* online). The instability of flesh color represents a major problem that must be overcome for the production of highly pure hybrids, but the mechanism by which it arises is unclear.

The objective of this study was to clarify the mechanisms underlying the accumulation of anthocyanins in the taproot of red-fleshed radish and the formation of the white-fleshed mutant. We mapped a transcription factor gene, *RsMYB1*, by using quantitative trait location by genome resequencing (QTL-seq) mapping and transcriptome analyses. We analyzed the mechanism that mediates the formation of the white-fleshed mutant and suggest that a *CACTA* transposon-induced methylation of the *RsMYB1* promoter might be associated with the production of white-fleshed mutants.

## Materials and methods

### Plant materials

Plant materials included the inbred line MTH01, which originated from the Xinlimei landrace and has red flesh and green skin, and JC01, which is a white-fleshed mutant derived from MTH01. An F_2_ segregating population was established from a cross between MTH01 and JC01 for gene mapping. A total of 646 F_2_ individuals were planted in a field at the Beijing Vegetable Research Center on 7 August 2015.

To determine the distribution of the *RsMYB1-CACTA* gene, a set of 111 radish cultivars with red, light green, or white flesh was used for analyzing haplotypes (Supplementary Table S2). Most of the samples were landraces obtained from the vegetable gene bank of the Beijing Vegetable Research Center. Total DNA was extracted from young leaves with a modified cetyltrimethylammonium bromide (CTAB) method (Murray and Thompson 1980).

### HPLC analysis of anthocyanins

Anthocyanins in the taproot skin and flesh were extracted and analyzed as described by Lin and Harnly (2007) with minor modifications. Freeze-dried samples (200 mg) were treated with 30 ml acidified methanol (0.1% HCl, v/v) and ultrasonication for 30 min at room temperature. A 10 μl aliquot of the solution was injected into an Eclipse XDB C18 column (4.6 mm×250 mm, 5 μm; Agilent Technologies, USA) and separated with 5% formic acid and acetonitrile:water:formic acid (50:45:5) as mobile phases on a 1290 series HPLC system (Agilent Technologies).

### Identification of QTLs related to red flesh in radish

To identify candidate genes related to anthocyanin biosynthesis, a QTL-seq analysis was performed as described by Takagi *et al.* (2013). Briefly, total DNA was extracted from young leaves of parents and each individual of F_2_ populations using the modified CTAB method (Murray and Thompson, 1980). Two DNA pools, red-fleshed bulk (MTH01 type) and white-fleshed bulk (JC01 type), were constructed by mixing an equal amount of DNA from 30 red-fleshed or 30 white-fleshed F_2_ individuals. Sequencing libraries from MTH01, JC01, and the two bulks were analyzed with the HiSeq 2500 platform (Illumina) (100 bp paired-end reads) at Annoroad Gene Technology Co., Ltd, Beijing, China. The clean reads for the red-fleshed bulk and the white-fleshed bulk were aligned to the ‘Aokubi DH’ (Mitsui *et al.*, 2015) and ‘XYB36-2’ (Zhang *et al*., 2015) reference genomes by using the BWA program (Li and Durbin, 2009). The GATK program (McKenna, *et al.*, 2010) was used for calling single nucleotide polymorphisms (SNPs) via the local reassembly of haplotypes for populations. We calculated the SNP-index for all variants. A sliding window analysis was completed with a 1 Mb window at a step size of 10 kb to calculate the mean SNP-index. The Δ(SNP-index) was calculated by subtracting the SNP-index of the red-fleshed bulk from that of the white-fleshed bulk. A 0.05 confidence level was applied as the threshold for detecting candidate QTL regions.

### Competitive allele-specific PCR genotyping and association mapping of candidate genes

To verify the correlation between the red-fleshed trait and SNP markers in the candidate QTL region, competitive allele-specific PCR genotype-specific primers designed based on the 100 bp flanking sequence were synthesized (Supplementary Table S3) and used for genotyping as described by Saxena *et al.* (2012).The parents and 646 F_2_ individuals were screened for co-segregating SNP markers, after which the JoinMap 4.0 program was used to generate a linkage map with a minimum logarithm of odds score of 4.0 (Van Ooijen, 2006).

### RNA-seq analyses

Tissue samples of mature MTH01 and JC01 taproots were collected and immediately frozen in liquid nitrogen and stored at −80 °C. Total RNA was extracted with the MiniBEST Plant RNA Extraction Kit (Takara, Japan). Sequencing libraries were generated with the NEBNext Ultra RNA Library Prep Kit for Illumina (NEB, USA). Index codes were added to attribute sequences to each sample. The libraries were sequenced with the HiSeq 4000 platform (Illumina) (150 bp paired-end reads) at Annoroad Gene Technology Co., Ltd, Beijing, China. Clean reads were aligned to the Aokubi DH reference genome (Mitsui *et al.*, 2015) by using the TopHat (Trapnell *et al.*, 2009) and Bowtie2 (Langmead *et al.*, 2009) programs. Gene expression levels were calculated based on the reads per kilobase of million mapped reads (RPKM) (Wagner *et al.*, 2012). The DEGseq program (Wang *et al.*, 2010) was used to identify differentially expressed genes (DEGs) based on the following criteria: |log_2_ ratio|≥1 and q<0.05. For the functional annotation of the related DEGs, refer to the previous study by Mitsui *et al.* (2015). The subsequent RNA-seq and quantitative reverse transcription (qRT)–PCR analyses were completed with two and three biological replicates, respectively.

### qRT–PCR analyses

The PrimeScript RT reagent kit with gDNA Eraser (Takara, Japan) was used to synthesize cDNA. A qRT–PCR assay was performed in a 20 μl reaction volume containing 100 ng cDNA, 10 μl 2× TB Green Premix Ex Taq II (Tli RNaseH Plus) (Takara), and 0.4 μM of each gene-specific primer. The PCR amplification was conducted in a LightCycler 480 System (Roche) with the following program: 95 °C for 30 s; 40 cycles of 95 °C for 5 s and 60 °C for 20 s; and then a melting curve analysis with one cycle of 95 °C for 5 s, 60 °C for 1 min, and 50 °C for 30 s. The data were analyzed with LightCycler 480 software (version 1.5; Roche) and the expression-level fold changes (relative to the 26S rRNA gene expression data) were calculated by using the 2^*-*ΔΔCt^ method. Details of the qRT–PCR primers are listed in Supplementary Table S3.

### Cloning of the coding and upstream regions of *RsMYB1-CACTA* in MTH01 and JC01

Primers for the PCR amplification of the *RsMYB1-CACTA* coding and upstream (−10 kb to −1 bp) regions were designed based on the corresponding *RsMYB1* (KR706195) and upstream sequences in the XYB36-2 reference genome. Specifically, the 10 kb region upstream of *RsMYB1-CACTA* was divided into four partially overlapping fragments for PCR amplification. New primers were then designed for a second PCR amplification, with the PCR products from the first amplification serving as templates. The resulting PCR fragments were sequenced and assembled with the DNAStar program. Details of the primers and the expected size of the amplified fragments are listed in Supplementary Table S3. The PCR was performed in a 50 μl reaction volume containing 100 ng DNA, 5 μl 10× PCR buffer (MgCl_2_), 1 μl of each primer (10 μM), 4 μl dNTPs (10 mM), 2.0 U TaqDNA polymerase, and double distilled H_2_O (Biomedical Technology Co., Beijing, China). The PCR amplification was conducted in a MyCycler system (Bio-Rad Laboratories, USA) with the following program: 95 °C for 2 min; 35 cycles of 95 °C for 30 s, appropriate annealing temperature for 30 s, and 72 °C for 1–4 min; and 72 °C for 10 min. The amplified fragments were purified and ligated into the pMD18-T vector. The resulting recombinant plasmids were inserted into *Escherichia coli* (DH5α) competent cells and then sequenced (Sangong, Shanghai, China).

### McrBC–PCR and bisulfite sequencing analysis

McrBC–PCR analysis was performed to estimate the degree of methylation of specific sequences. Genomic DNA (1 μg) extracted from mature radish taproot flesh was digested overnight with the methylation-specific restriction endonuclease McrBC (NEB, USA) according to the manufacturer’s instructions. The digested DNA was used as the template for a PCR, which was conducted with two biological replicates. The *RsMYB1-CACTA* promoter and upstream sequences were divided into the following two fragments: transposon region (−1789 to −1009 bp) and promoter region (−303 to −46 bp). The fragments were then amplified by PCR with fragment-specific primers (Supplementary Table S3). The amount of amplification product was used to estimate the degree of methylation of the corresponding region.

For bisulfite sequencing (BS-seq) analysis, 500 ng of genomic DNA extracted from mature radish taproot flesh was treated with sodium bisulfite using the EZ DNA Methylation-Gold Kit (Zymo Research, USA). The following five fragments upstream of *RsMYB1* were amplified by a temperature-gradient PCR program with bisulfite-treated DNA and the respective degenerate primers (Supplementary Table S3): BS1 (−1842 to −1648 bp), BS2 (−1578 to −1331 bp), BS3 (−1294 to −1024 bp), BS4 (−581 to −424 bp), and BS5 (−176 to −1 bp). The PCR products were purified and then cloned into the pMD18-T vector. To accurately estimate the degree of methylation of specific sequences, 10 clones were sequenced (Sangong, Shanghai, China). The ratio of C-methylation was calculated as the number of methylated bases divided by the total number of bases at each cytosine site.

### Treatment with 5-azacytidine

The cytosine analog 5-azacytidine (5-azaC) is a demethylating agent that can influence plant development. Seeds of the white-fleshed mutant JC01 were treated with various concentrations of 5-azaC [0 mM (control), 1 mM, 10 mM, and 20 mM]; 1000 seeds were treated with each concentration. Briefly, surface-sterilized seeds were placed in 10 Petri dishes containing filter paper moistened with the 5-azaC solution and incubated at 20 °C for 6 days. The filter paper was replaced every 2 days. The resulting seedlings were transplanted into 100-hole trays and grown in a greenhouse in natural conditions, after which the phenotypes of the seedlings were analyzed. Additionally, seedling samples were collected and stored at −80 °C for subsequent anthocyanin extraction and qRT–PCR analysis.

### Virus-induced gene silencing

A tobacco rattle virus (TRV)-based virus-induced gene silencing (VIGS) system was used to functionally characterize *RsMYB1*. A small red radish, ‘Gangshuiluobo’ (No. 87 in Supplementary Table S2), which has an *RsMYB1* gene and CACTA transposon that is identical to that of the MTH01 *RsMYB1-CACTA* gene, was used in the VIGS experiment. A 488 bp *RsMYB1-CACTA* fragment was amplified and cloned into a pTRV2 vector to generate a pTRV2*-RsMYB1-CACTA* recombinant plasmid. Overnight cultures of *Agrobacterium tumefaciens* strain GV3101 cells containing pTRV1, pTRV2, or pTRV2*-RsMYB1-CACTA* were centrifuged (3000 *g* for 15 min), resuspended in infiltration buffer [10 mM MgCl_2_, 10 mM 2-(*N*-morpholino)ethanesulfonic acid, and 100 μM acetosyringone], and incubated at 28 °C for 3 h in darkness. The suspensions with cells carrying pTRV2 or pTRV2-*RsMYB1-CACTA* were mixed with the suspension with cells carrying pTRV1 at a 1:1 (v/v) ratio. The mixtures were infiltrated into the cotyledons of 8-day-old radish seedlings by using a 1 ml syringe without a needle (Liu *et al.*, 2002). The infiltrated plants were grown in a growth chamber at 22 °C and 60% relative humidity, with a 16 h light/8 h dark photoperiod. When fading of the red color in the leaves or stems appeared, an RT–PCR assay was conducted to detect the pTRV1 and pTRV2 plasmids in the leaves and stems. Plants carrying pTRV1 + pTRV2 or pTRV1 + pTRV2-*RsMYB1-CACTA* were examined to assess their *RsMYB1-CACTA* expression. Details of the primers used for this analysis are listed in Supplementary Table S3.

### Generation of *RsMYB1*-overexpressing transgenic calli

A *p35S:RsMYB1-CACTA-GFP* vector containing the full-length *RsMYB1-CACTA* coding sequence (without the termination codon) fused in-frame with the gene encoding green fluorescent protein (GFP) under the control of the 35S promoter was constructed. Details of the primers used to generate this construct are listed in Supplementary Table S3. To produce *RsMYB1-CACTA*-overexpressing transgenic lines, *A. tumefaciens* strain GV3101 cells carrying *p35S:RsMYB1-CACTA-GFP* were used for transformations, which were conducted as described by Cho *et al.* (2008). However, no shoots were regenerated from the JC01 explants. Calli were examined with a fluorescence microscope (Leica Microsystem, Heidelberg, Germany).

## Results

### Phenotypic characterization of MTH01 and JC01

**Fig. 1.**
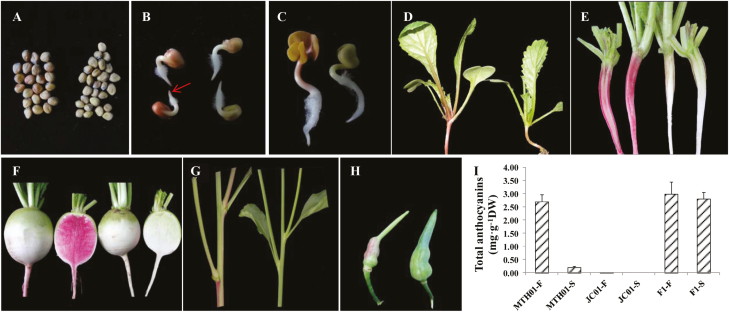
Phenotypic comparison of MTH01 (left) and JC01 (right). (A) Seeds. (B) Radicles emerging from germinated seeds. The arrow indicates the accumulation of red pigment at the radicle tip. (C) Cotyledons and hypocotyls. (D) 10-day-old seedlings. (E) 3-week-old roots. (F) 7-week-old roots. (G) Flowering branches. (H) Siliques. (I) Total anthocyanin levels in the root flesh and skin of MTH01, JC01, and F_1_ hybrid plants. Data presented are the mean ±SD from three individuals. DW, dry weight; F, flesh; S, skin. (This figure is available in colour at *JXB* online.)

Both the red-fleshed wild-type and the white-fleshed mutant radish plants grew and developed normally under normal field conditions. In MTH01, anthocyanins accumulated in the taproot flesh as well as in other tissues, including part of the seed coat, the radicle tips emerging from germinated seeds, the cotyledons and hypocotyls, young seedlings, the root of 3-week-old plants, the lateral branch of flowering plants, and the siliques (Fig. 1). The color of the root skin gradually faded from the cortex splitting stage to the mature stage in MTH01 (Fig. 1E, F). Under the same growth conditions, anthocyanin accumulation was not observed in the taproot flesh or in almost all analyzed tissues of the mutant JC01 (Fig. 1A–C, F–H). The exceptions were the hypocotyls at the seedling stage and the root skin at the cortex splitting stage (Fig. 1D, E).

The anthocyanin contents of the taproot flesh and skin of MTH01, JC01, and F_1_ hybrid (MTH01 × JC01) plants were determined by HPLC analysis. High anthocyanin contents were detected in the root flesh of MTH01 (2.71 mg g^–1^ dry weight) and the root flesh and skin of the F_1_ hybrid (3.01 and 2.81 mg g^–1^ dry weight, respectively) (Fig. 1I). In contrast, the anthocyanin content in the MTH01 root skin was low (0.2 mg g^–1^ dry weight). Moreover, anthocyanins were almost undetectable in the root flesh of JC01 (0.01 mg g^–1^ dry weight), implying that the anthocyanin pathway was inhibited in JC01 under normal growth conditions.

### The white-fleshed mutant is the result of a single recessive gene

**Fig. 2.**
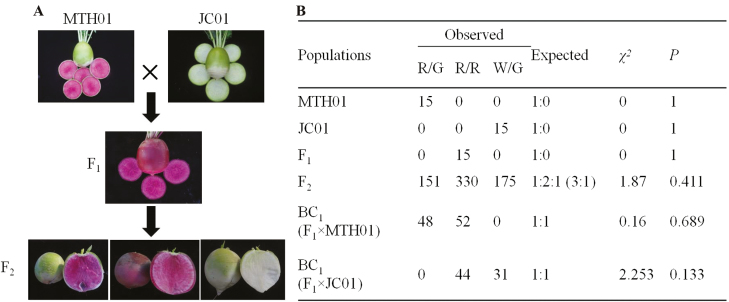
Phenotype and inheritance patterns of the white-fleshed mutant. (A) Root flesh and skin colors in the MTH01, JC01, and F_1_ and F_2_ hybrid plants. (B) Inheritance patterns of the white-fleshed mutant in segregating populations from the MTH01 × JC01 cross. R/G, red flesh and green skin; R/R, red flesh and red skin; W/G, white flesh and green skin. (This figure is available in colour at *JXB* online.)

To examine the inheritance of the mutation resulting in white taproot flesh, we crossed MTH01 with JC01, and the generated heterozygous F_1_ plants were self-crossed and backcrossed with parental lines to produce F_2_ and BC_1_ populations, respectively. Intense red coloration was observed in the taproot skin and flesh of all F_1_ plants, even though both parents have green root skin (Fig. 2A). The phenotypes of the F_2_ and BC_1_ plants were investigated in terms of the absence or presence of red flesh and red skin. For the F_2_ population, the red flesh and green skin:red flesh and red skin:white flesh and green skin segregation ratio was 1:2:1 (*χ*^2^=1.87, *P*=0.411). For the two BC_1_ populations, the red flesh and red skin:white flesh and green skin segregation ratio was 1:1 (*χ*^2^=2.253, *P*=0.133; Fig. 2B). These results implied that the white-fleshed trait of the mutant was controlled by a single recessive gene (designated *red1*).

### *RsMYB1* is a candidate gene responsible for anthocyanin accumulation in red-fleshed radish

**Fig. 3.**
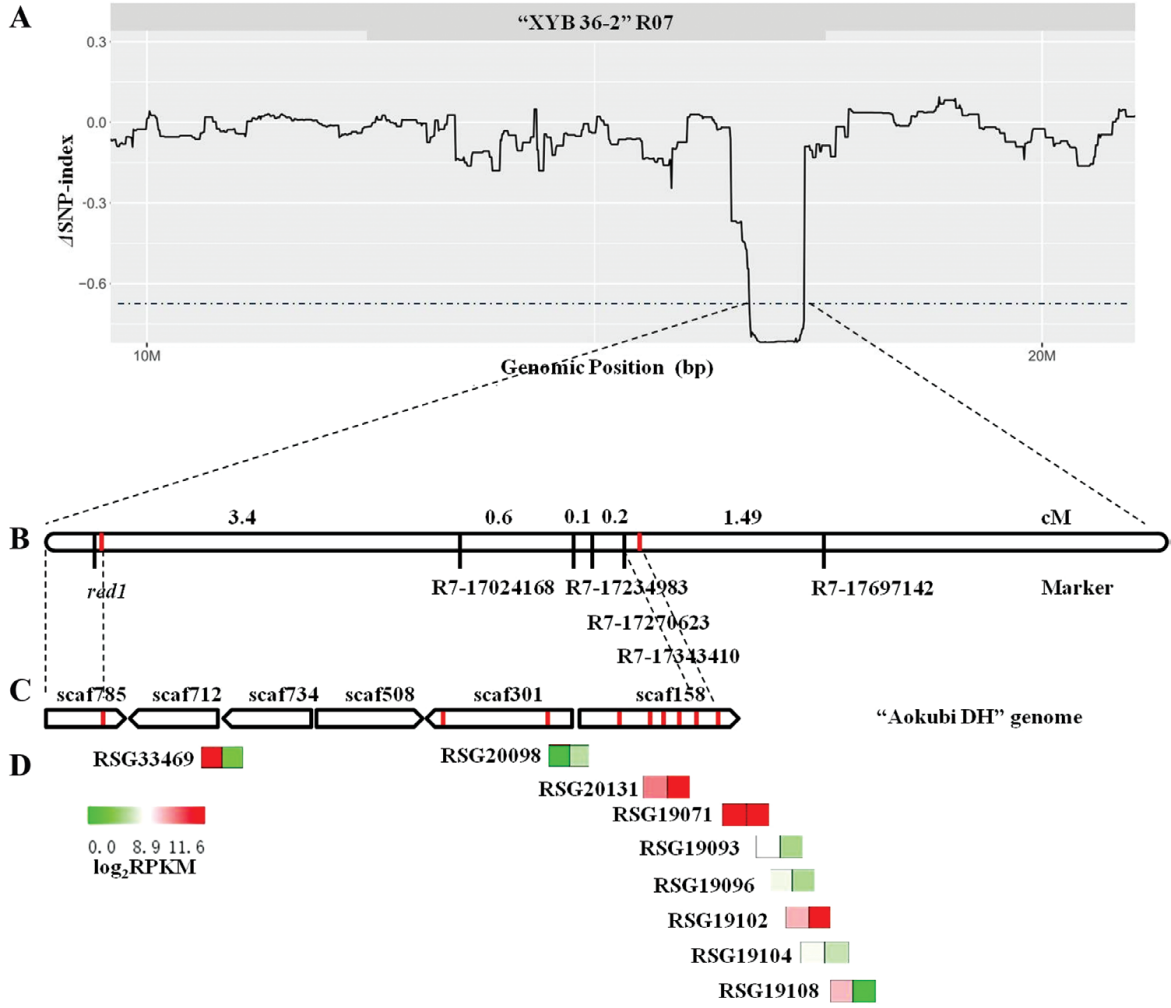
Identification of *RsMYB1* as a candidate gene responsible for anthocyanin accumulation in red-fleshed radish based on QTL-seq and RNA-seq analyses. (A) Δ(SNP-index) graph from the QTL-seq analysis. The x-axis represents the positions on chromosome R07 and the y-axis represents the Δ(SNP-index). A candidate QTL was identified on chromosome R07 (16.25–18.33 Mb interval), with a Δ(SNP-index) greater than 0.7 (*P<0.05*). (B) Genetic distances of SNP markers and a candidate gene (*red1*) on chromosome R07 (16.25–18.33 Mb interval) according to linkage analyses involving 646 F_2_ individuals. (C) Comparison of the syntenic regions between chromosome R07 (16.25–17.35 Mb interval) from the XYB36-2 genome and six scaffolds from the Aokubi DH genome based on an online BLAST analysis. (D) Differentially expressed genes (indicated by the vertical bars in panel C) in the candidate region and their relative expression levels based on RNA-seq data. The heatmap was prepared with the MeV4.9.0 program: left, MTH01; right, JC01. (This figure is available in colour at *JXB* online.)

A candidate gene responsible for the radish taproot flesh color was detected based on a QTL-seq strategy. A total of 24.6 Gb and 24.53 Gb clean bases was obtained for the red-fleshed and white-fleshed bulks, respectively, and the genome coverage was 60.52-fold and 59.47-fold, respectively (referring to the XYB36-2 genome) (Supplementary Table S4). A total of 79 831 SNPs were identified on nine chromosomes. Graphs indicating the relationship between the SNP-index and chromosome positions were generated for the red-fleshed and white-fleshed bulks. A Δ(SNP-index) graph (plotted against genome positions) was also prepared (Supplementary Tables S5 and S6, Supplementary Fig. S1). The *red1* locus was localized to a 2.08 Mb region (16.25–18.33 Mb interval) on chromosome 7 containing 1285 SNP loci (Fig. 3A). Further linkage analyses revealed that the R7-17024168 marker was the closest SNP to *red1* (genetic distance 3.4 cM) in 646 individuals from the F_2_ population (Fig. 3B). Additional markers closely linked to *red1* were not identified within the anchoring interval. A QTL-seq analysis based on another reference genome (that of Aokubi DH) was conducted, resulting in the detection of 16 candidate regions, covering 281 186 bp, with 153 SNPs (Supplementary Table S7). Six scaffolds from the Aokubi DH genome, including the Rs_scaf158: 166 979–271 436 and Rs_scaf301: 22 876–32 892 regions, were anchored to the candidate region via an online BLAST analysis (Fig. 3C).

To investigate the DEGs and the anthocyanin biosynthetic or regulatory genes in the candidate region, we compared the transcriptomes of MTH01 and JC01. A total of 796 DEGs were identified, including 335 up-regulated and 461 down-regulated genes (Supplementary Table S8). A KEGG analysis was completed to identify the significantly enriched pathways associated with the DEGs. Seven enriched pathways were related to biosynthesis of secondary metabolites, including flavonoid biosynthesis (map00941) and anthocyanin biosynthesis (map00942) (Supplementary Table S9). In the candidate region, 183 genes were annotated in six scaffolds of the Aokubi DH genome, including nine genes that were differentially expressed between MTH01 and JC01 (Supplementary Table S10, Fig. 3D). Among these DEGs, RSG33469 (homologous gene Rsa10033919, located on XYB36-2 chromosome R07: 16 320 148) and RSG19108 (homologous gene Rsa10034073, located on XYB36-2 chromosome R07: 17 352 870) were annotated as the anthocyanin synthesis regulator gene *AtMYB90* (*PAP2*), which encodes an R2R3-MYB transcription factor family protein (Fig. 3B, D). Thus, RSG33469 and RSG19108 were named *RsMYB1* and *RsMYB2*, respectively.

The coding sequence of *RsMYB1* in MTH01 was isolated by RT–PCR; *RsMYB2* was not amplified. A sequence alignment analysis revealed 1–3 SNPs between the MTH01 *RsMYB1* coding sequence and the sequence of *RsMYB1* in the analyzed reference genomes, as well as 26 SNPs in a comparison with *RsMYB2* (Supplementary Table S11). This confirmed that only *RsMYB1* was expressed in the MTH01 taproot flesh. Although the transcriptome data (RPKM analysis) appeared to indicate that *RsMYB2* was expressed (Fig. 3D), this may have been a false-positive result due to the considerable similarity between *RsMYB1* and *RsMYB2*. The results indicated that *RsMYB1* is most likely the *red1* gene.

### A transposon-induced methylation of the *RsMYB1* promoter results in down-regulated expression in white-fleshed mutants

**Fig. 4.**
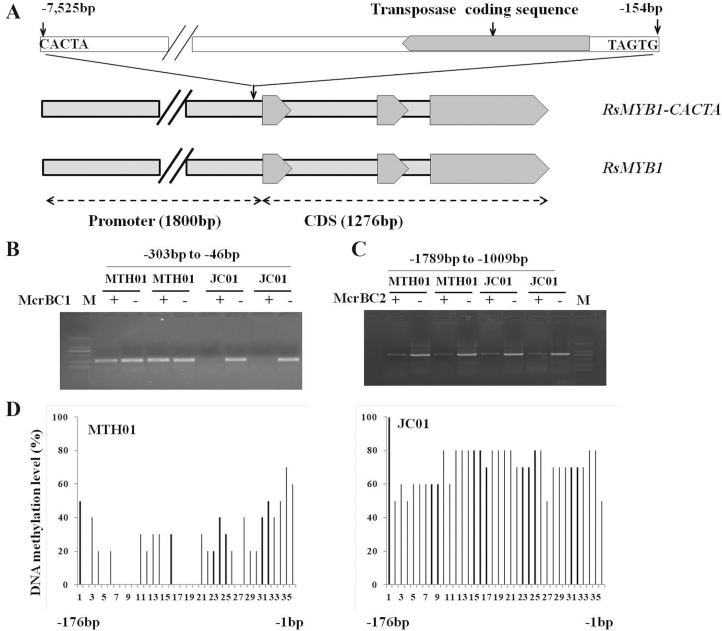
Analysis of cytosine methylation of the *RsMYB1* promoter region in MTH01 and JC01. (A) Structural representation and localization of the CACTA transposon inserted at the *RsMYB1* locus. (B, C) McrBC-sensitive PCR analysis of the *RsMYB1-CACTA* promoter region (−46 to −303 bp) and the CACTA transposon region (−1009 to −1789 bp) in MTH01 and JC01. + and − indicate whether genomic DNA was treated with McrBC before PCR amplification. The absence of a PCR product in the McrBC-treated samples indicates the DNA was methylated. M, marker DL2000. (D) Analysis of cytosine methylation of the promoter region (BS5; −176 to −1 bp relative to the *RsMYB1-CACTA* start codon, ATG) by bisulfite sequencing (location information from Supplementary Fig. S2A). The percentage of methylation of each cytosine in the region is indicated by the vertical bars.

The promoter and coding region of the MTH01 and JC01 *RsMYB1* genes was isolated by long-range PCR and chromosome walking (GenBank ID: MN308185). An analysis of the nucleotide sequences revealed a lack of differences between MTH01 and JC01. However, the insertion of a CACTA transposon from the En/Spm family, containing a terminal inverted repeat (5′-*CACTA*CAAGAAAA-3′) (Tian, 2006), was identified at position −154 bp upstream of *RsMYB1* in MTH01 and JC01 (Fig. 4A). Hereafter, we use *RsMYB1-CACTA* to represent the allele of *RsMYB1* from MHT01 and JC01.

Transposable elements are widely distributed in the genome and affect the expression of linked genes, notably via the spreading of DNA methylation (Martin *et al.*, 2009; Wittmeyer *et al.*, 2018). Methylation of the *RsMYB1-CACTA* promoter might down-regulate the expression of *RsMYB1-CACTA*. We examined the extent of DNA methylation of the *RsMYB1-CACTA* promoter (−303 to −46 bp) in MTH01 and JC01 with the methylation-specific enzyme McrBC and observed that the *RsMYB1-CACTA* promoter of JC01 was highly methylated (Fig. 4B). However, the high level of DNA methylation in the transposon region (−1789 to −1009 bp) upstream of the promoter was not significantly different between MTH01 and JC01 (Fig. 4C).

We also compared the level of cytosine methylation in the promoter (BS5) and transposon (BS1, BS2, BS3, and BS4) regions of the *RsMYB1-CACTA* genes in MTH01 and JC01 based on a quantitative BS-seq analysis of genomic DNA extracted from taproot flesh (Supplementary Fig. S2B). The results confirmed the hypermethylation of the *RsMYB1-CACTA* promoter in the JC01 mutant, in which 70.56% of the cytosines were methylated. In contrast, only 22.77% of the cytosines were methylated in MTH01 (Fig. 4D, Supplementary Fig. S2B). Regarding the transposon regions, BS1 (−1842 to −1648 bp), BS2 (−1578 to −1331 bp), BS3 (−1294 to −1024 bp), and BS4 (−581 to −424 bp) were highly methylated in both MTH01 and JC01, with cytosine methylation rates of 62.33–90.7%. There were no significant differences between MTH01 and JC01 in the methylation of BS2, BS3, and BS4, but BS1 was more highly methylated in MTH01 than in JC01 (Supplementary Fig. S2B, C).

The hypermethylation of the *RsMYB1-CACTA* promoter was also confirmed in various new chimeric mutants, reinforcing the correlation between the high level of DNA methylation of the *RsMYB1-CACTA* promoter and the production of a white-fleshed taproot in MTH01. In the MTH01 chimeric mutants M1, M2, and M3, the level of methylation of the *RsMYB1-CACTA* promoter was considerably higher in the white-fleshed part of the taproot than in the red-fleshed part (Supplementary Fig. S3). In summary, the cytosines of the CACTA transposon were highly methylated and the DNA methylation had spread to the *RsMYB1* promoter, resulting in the inhibition of expression of this gene and the production of a white-fleshed taproot in JC01. This finding also explained the high frequency of white-fleshed mutants in MTH01.

### Functional complementation of the taproot flesh color phenotype

**Fig. 5.**
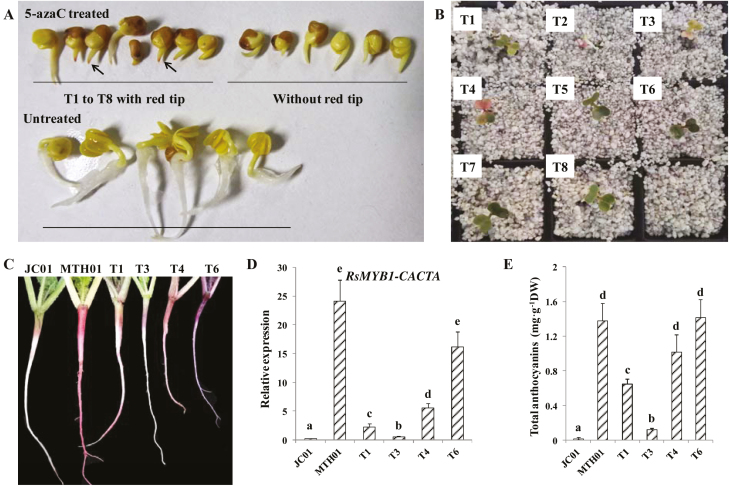
Treatment with 5-azaC reversed the mutant phenotype of JC01, likely as a result of demethylation. (A) Phenotypes of 5-azaC-treated individuals (T1–T8) and untreated controls at the germination stage. Arrows indicate the accumulation of red pigment at the radicle tip. (B, C) Phenotypes of 5-azaC-treated individuals at the cotyledon expansion and young seedling stages. (D) Relative expression level of *RsMYB1-CACTA* in JC01, MTH01, and the 5-azaC-treated samples, based on a qRT–PCR analysis. (E) Total anthocyanin levels in the root flesh and skin of JC01, MTH01, and 5-azaC-treated samples, based on an HPLC analysis. DW, dry weight. In (D) and (E), data presented are the mean ±SD from three technical replicates and different letters indicate significant differences (*P*=0.05). (This figure is available in colour at *JXB* online.)

To further verify that the down-regulated expression of *RsMYB1-CACTA* resulted from methylation of the promoter, JC01 seeds were treated with different concentrations of the demethylating agent 5-azaC (500 μM, 1 mM, and 5 mM). The mutant phenotype was reversed in eight individuals treated with 1 mM 5-azaC. Specifically, a red pigment was observed at the tip of radicles emerging from germinated seeds in the 5-azaC-treated mutants at 6 days after the treatment (Fig. 5A). At 2 weeks after sowing, three of these 5-azaC-treated mutants accumulated anthocyanins in the cotyledons (Fig. 5B), and four mutant seedlings were obtained (T1, T3, T4, and T6; Fig. 5C). T1 exhibited discontinuous accumulation of anthocyanin in the roots. In T3, a red pigment accumulated mainly in the hypocotyl, with no or only very small amounts of pigment in the roots. In contrast, in T4 a red pigment accumulated throughout the seedling, including the roots and petioles. T6 accumulated a purple pigment. An HPLC analysis revealed the increased anthocyanin contents in the roots of the four mutant seedlings (Fig. 5E). The up-regulated expression of *RsMYB1-CACTA* was also confirmed (Fig. 5D). These results implied that the demethylation by 5-azaC activated *RsMYB1-CACTA* expression, thereby enhancing anthocyanin accumulation in the white-fleshed JC01 mutant.

**Fig. 6.**
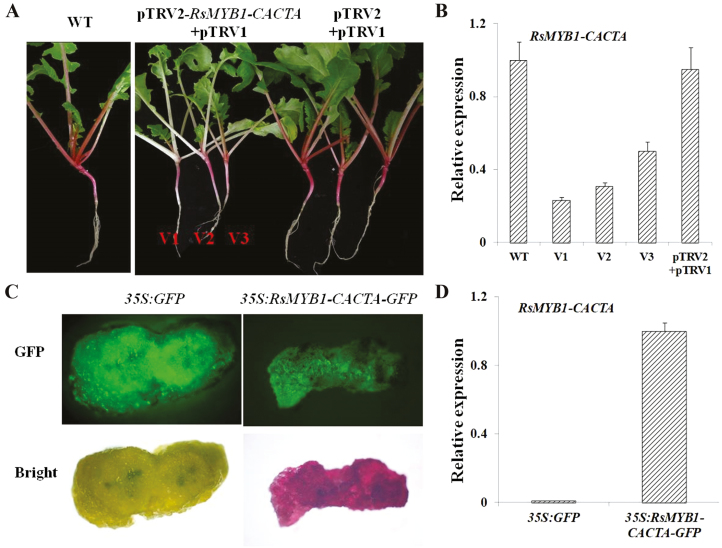
Functional complementation analysis of *RSMYB1-CACTA* by VIGS and *Agrobacterium* transformation. (A) Seedlings injected with pTRV2-*RsMYB1-CACTA* + pTRV1 or pTRV2 + pTRV1. WT, wild-type control. (B) Relative expression of *RsMYB1-CACTA* in the petioles collected from seedlings injected with pTRV2-*RsMYB1-CACTA* + pTRV1 (V1, V2, and V3) or pTRV2 + pTRV1 (mean ±SD, *n*=3). (C) Anthocyanin accumulation in *35S:GFP* and *35S:RsMYB1-CACTA-GFP* transgenic JC01 radish calli. (D) Relative *RsMYB1-CACTA* expression in the transgenic calli (mean ±SD, *n*=3). (This figure is available in colour at *JXB* online.)

VIGS may lead to post-transcriptional gene silencing. The pTRV2*-RsMYB1-CACTA* recombinant plasmid was used to silence *RsMYB1-CACTA* expression in the radish Gangshuiluobo, which has red skin. At 2 weeks after the infiltration, the petioles and root skin of control seedlings remained red, whereas the seedlings infiltrated with pTRV2-*RsMYB1-CACTA* had white or light red petioles and root skin (Fig. 6A). Additionally, the expression level of *RsMYB1-CACTA* was lower in the seedlings infiltrated with pTRV2-*RsMYB1-CACTA* than in the control plants (Fig. 6B). These results suggested that *RsMYB1-CACTA* is important for anthocyanin accumulation in radish. Furthermore, the overexpression of *RsMYB1-CACTA* in the JC01 mutant resulted in the production of red transgenic calli (Fig. 6C, D).

### The CACTA transposon near *RsMYB1* is essential for the red-fleshed phenotype of radish

**Fig. 7.**
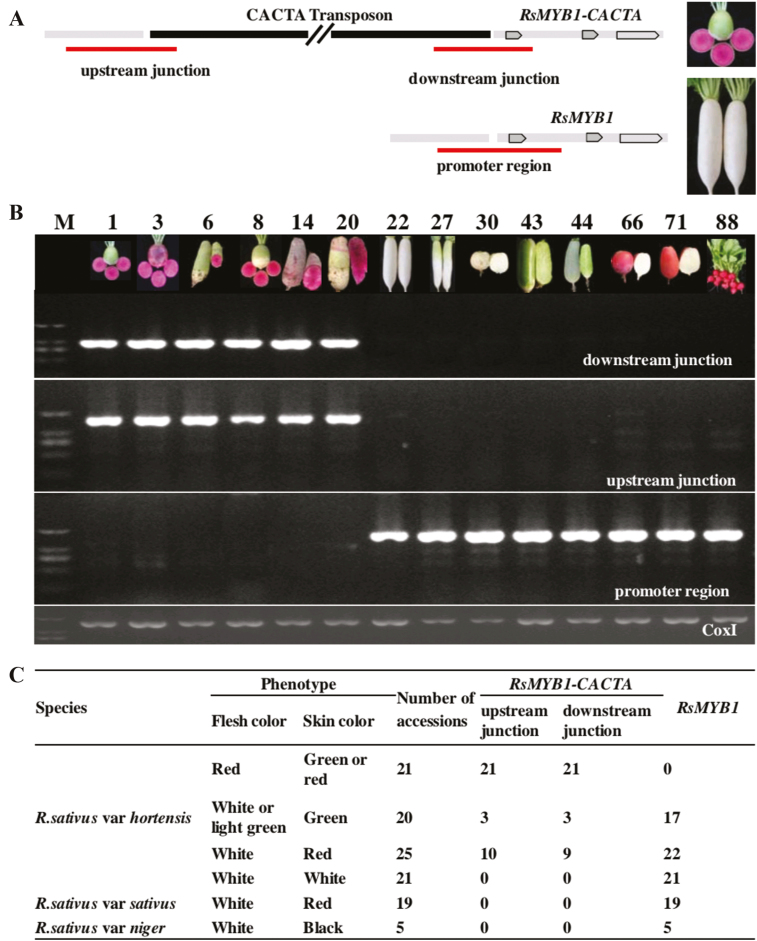
The phenotype of red-fleshed radish is associated with the insertion of the CACTA transposon in the promoter region of *RsMYB1*. Molecular structures of *RsMYB1-CACTA* and *RsMYB1* alleles with flanking sequences are presented. (A) The insertion sites upstream of *RsMYB1-CACTA* and *RsMYB1* are indicated for a red-fleshed line (MTH011) and a white-fleshed line (Baiyuchun). (B) Images of 14 radish varieties that varied in terms of flesh color, and results of agarose gel electrophoresis for the PCR-based analysis of the *RsMYB1-CACTA* downstream and upstream junctions and the *RsMYB1* promoter region of each variety. *Cox1* served as a reference gene. The 1000 bp and 1800 bp fragments corresponding to the *RsMYB1-CACTA* downstream and upstream junctions are present in the red-fleshed varieties (lanes 1–6), but not in the non-red-fleshed varieties (lanes 7–14). (C) PCR-based analysis of the inserted CACTA transposon in 111 accessions. The data presented in (B) and (C) are provided in Supplementary Table S2. (This figure is available in colour at *JXB* online.)

The CACTA transposon inserted in the *RsMYB1* promoter region was identified in a natural population. We screened 111 radish accessions varying in skin and flesh colors collected from various regions (Fig. 7, Supplementary Table S2). Our results revealed that all 21 tested red-fleshed accessions carried the CACTA transposon. Moreover, the transposon was not detected in 21 white-skinned and white-fleshed accessions, 19 European small radish accessions with red skin and white flesh, and 5 black radish accessions with black skin and white flesh. However, 3 of 20 green-skinned radishes (with white or light green flesh) harbored the CACTA transposon. It was not clear whether the *RsMYB1-CACTA* promoter in these accessions was methylated as in JC01 (which has green skin). Surprisingly, of the 25 East Asian radish accessions with red skin, 10 accessions contained the CACTA transposon and 15 accessions contained the common *RsMYB1* promoter; 7 accessions were heterozygous. The fact that all of the tested red-fleshed accessions contained the CACTA transposon implied that the CACTA transposon in the *RsMYB1* promoter region is required for the formation of red-fleshed taproot.

## Discussion

The appearance of white-fleshed mutants in red-fleshed radish, which severely affects seed purity, has puzzled breeders for years. In this study, QTL-seq and transcriptome sequencing techniques were used and identified *RsMYB1-CACTA* as an important gene involved in anthocyanin accumulation in red-fleshed radish. Sequence analysis revealed a 7372 bp CACTA transposon in the *RsMYB1* promoter region. However, we did not detect any sequence differences in the *RsMYB1-CACTA* coding and regulatory regions between red-fleshed and white-fleshed lines. Further analyses involving McrBC and BS-seq indicated that the extensive methylation of the *RsMYB1-CACTA* promoter region may be due to the spreading of DNA methylation from the nearby transposon region, ultimately resulting in the down-regulation of *RsMYB1-CACTA* expression and the formation of white-fleshed radish taproots. This type of epigenetic change may explain the occasional appearance of white-fleshed mutants in commercially bred red-fleshed radish.

### *RsMYB1* is a key gene for anthocyanin accumulation in red-fleshed radish

Previous studies have combined QTL-seq and RNA-seq analyses to identify candidate genes (Wittmeyer *et al.*, 2018; Park *et al.*, 2019). In the current study, although the closest marker linked to *red1* was R7-17024168 (genetic distance 3.4 cM), nine genes near this region were differentially expressed between MTH01 and JC01, of which RSG33469 (*RsMYB1*) and RSG19108 (*RsMYB2*) were R2R3-MYB-type anthocyanin-regulating genes. A linkage map and an analysis of the physical distances for *red1*, *RsMYB1*, and *RsMYB2* (Fig. 3B, C) based on two reference genomes indicated that *RsMYB1* is most likely the *red1* gene. We therefore identified *RsMYB1* as the gene responsible for anthocyanin accumulation in red-fleshed radish. This gene (GenBank ID: KR706195) was first cloned by Lim *et al.* (2016) from a red-fleshed radish line. The *RsMYB1* and *RsMYB2* genes from MTH01 and three reference genomes were clearly divided into two groups in a phylogenetic tree (Supplementary Fig. S4). A sequence alignment analysis revealed 1–3 SNPs between the MTH01 *RsMYB1* coding sequence and the *RsMYB1* coding sequence in the analyzed reference genomes, as well as 26 SNPs in a comparison with *RsMYB2* (Supplementary Table S11). This confirmed that only *RsMYB1* was expressed in the taproot flesh of MTH01. Although the transcriptome data appeared to indicate that *RsMYB2* was expressed, this may have been a false-positive result due to the considerable similarity between *RsMYB1* and *RsMYB2*. The function of *RsMYB1-CACTA* was further confirmed by overexpression in transgenic radish.

In previous reports, Yi *et al.* (2018) and Luo *et al.* (2020) suggested that *RsMYB2* (Rs388430, ‘WK10039’ R07: 9.36 Mb) is a key gene for anthocyanin accumulation in red radish skin. However, SNPs in the *RsMYB2* coding region and promoter region were not well correlated with the red-skin phenotype in the natural population, implying that there is more than one locus controlling the production of red skin (Tatebe, 1938; Harborne and Paxman, 1964). Moreover, an intense red coloration was observed in the taproot skin and flesh of all F_1_ plants in this study, although both parents had green root skin (Fig. 2A). The mechanism underlying anthocyanin accumulation may differ between the root skin and flesh. Furthermore, whether *RsMYB1* and *RsMYB2* have synergistic effects needs to be investigated.

### A CACTA transposon-induced DNA methylation of the *RsMYB1* promoter is associated with the white-fleshed mutation in red-fleshed radish

The *MYB* transcription factors are important regulators of anthocyanin biosynthesis. The color of plant organs may change in response to naturally occurring mutations of *MYB* genes, including SNPs and insertions/deletions (Ban *et al.*, 2007, Espley *et al.*, 2009), as well as the insertion of transposons (Kobayashi *et al.*, 2004; Zhang *et al.*, 2019) and methylation of the *MYB* promoter (Wang *et al.*, 2013). In the current study, we proved that the white-fleshed mutant is the result of an epigenetic change in the *RsMYB1-CACTA* promoter. This natural and heritable epigenetic change was due to the insertion of a CACTA transposon, which was hypermethylated, and the spreading of DNA methylation to the *RsMYB1* promoter (Fig. 4). In *Arabidopsis thaliana*, transposons and repeated sequences are usually heavily methylated in all three cytosine contexts (Zhang *et al.*, 2006; Lister *et al.*, 2008). Methylation of promoter DNA usually inhibits gene transcription, and is often the result of the spreading of DNA methylation from nearby transposons and other repeats (Zhang *et al.*, 2018). Meanwhile, ROS1-mediated demethylation helps to establish boundaries between transposons and genes, thereby preventing the spreading of DNA methylation and transcriptional silencing from transposons to neighboring genes (Tang *et al.*, 2016; Zhang *et al.*, 2018). Our McrBC and BS-seq analyses confirmed that the CACTA transposon region was hypermethylated in both MTH01 and JC01 (Supplementary Fig. S2). The fact that the CACTA transposon was inserted very close to *RsMYB1-CACTA* (−154 bp relative to the *RsMYB1-CACTA* start codon) likely explains the increased methylation of the *RsMYB1* promoter region and the associated down-regulation of gene expression (Supplementary Fig. S2). On the other hand, we speculate that the spreading of methylation from the highly methylated transposon in the red-fleshed line MTH01 might be suppressed by a ROS1-mediated mechanism, in line with similar research (Tang *et al.*, 2016; Zhang *et al.*, 2018).

The cytosine analog 5-azaC may strongly demethylate the genome in diverse plant systems, leading to growth retardation, malformations, and the reactivation of expression of silenced genes (Solís *et al.*, 2015; Tyč *et al.*, 2017; Zhu *et al.*, 2018). We found that demethylation of the *RsMYB1-CACTA* promoter by treatment with 5-azaC induced the accumulation of anthocyanins in the white-fleshed mutant JC01. Moreover, in the MTH01 chimeric mutants M1, M2, and M3, the *RsMYB1-CACTA* promoter was more extensively methylated in genomic DNA extracted from the white-fleshed taproot region than in genomic DNA from the red-fleshed taproot region (Supplementary Fig. S3). The underlying mechanism is similar to that regulating sex expression in melon, where the transition from male to female flowers in gynoecious lines occurs following the insertion of hAT family transposons, which results in the spreading of DNA methylation to the *CmWIP1* promoter (Martin *et al.*, 2009).

### *RsMYB1* regulates early anthocyanin biosynthetic pathway genes in red-fleshed radish

In *A. thaliana*, 35 anthocyanin biosynthetic genes (ABGs) have been identified (18 structural genes, 16 regulatory genes, and 1 transport gene) (Guo *et al.*, 2014). An analysis of syntenic homology uncovered 53 radish ABGs (Supplementary Table S12). The radish genome consists of more than one copy of most of these ABGs because of a whole-genome triplication event (Supplementary Table S12). Similarly, Sun *et al.* (2018) identified 44 structural and 182 transcription factor genes (*MYB*, *bHLH*, and *WD40* genes) associated with the anthocyanin biosynthetic pathway based on transcriptome data.

A comparison of the MTH01 and JC01 root flesh transcriptomes indicated that the expression levels of 27 of the 53 ABGs were considerably down-regulated. Specifically, the expression levels of at least one copy of the ABGs (*RsPAL*, *RsC4H*, *Rs4CL*, *RsCHS*, *RsCHI*, *RsF3H*, *RsDFR*, *RsANS*, and *RsUF3GT*) and a transport gene (*RsTT19*) were substantially down-regulated in the mutant (Supplementary Fig. S5). The down-regulated expression of these structural genes suggests that *RsMYB1* is a key gene in the regulatory network controlling anthocyanin biosynthesis. An earlier investigation in *A. thaliana* showed that the overexpression of *AtPAP1*, which encodes an R2R3-MYB transcription factor, up-regulates the expression of a series of anthocyanin and flavonol biosynthetic genes (Tohge *et al.*, 2005). The anthocyanin biosynthetic pathway structural genes regulated by the MYB-bHLH-WD40 complex are species- and tissue-specific. Previous studies indicated that the late pathway genes in *A*. *thaliana* (Borevitz *et al.*, 2000; Gonzalez *et al.*, 2008) and the early and late pathway genes in maize (Grotewold *et al.*, 1994; Falcone Ferreyra et al., 2010) are regulated by this complex. In the current study, the first anthocyanin biosynthetic pathway gene that exhibited dramatically down-regulated expression was *CHS*, with the expression levels of three copies down-regulated based on RNA-seq data (Supplementary Table S12, Supplementary Fig. S5). Therefore, *RsMYB1* may regulate the expression of the early anthocyanin biosynthetic pathway genes, beginning with *RsCHS*. However, the genes targeted by the MYB-bHLH-WD40 complex will need to be confirmed by chromatin immunoprecipitation.

### The CACTA transposon and the origin of red-fleshed radish

Earlier investigations proved that CACTA and other types of transposons function as enhancers that can influence the expression of nearby genes depending on their methylation status (Oikawa *et al.*, 2015; Wittmeyer *et al.*, 2018; Zhang *et al.*, 2019). A gypsy-like long terminal repeat retrotransposon inserted upstream of *MdMYB*1 is responsible for the red-skinned phenotype in apple (Zhang *et al.*, 2019). In maize, an inserted Hopscotch transposon serves as a long-distance enhancer of *tb1* expression and helps to alter apical dominance (Studer *et al.*, 2011). An 11.0 kb insertion positively influences the expression of *Kala4*, which is associated with the production of black rice (Oikawa *et al.*, 2015). Thus, we suggest that the CACTA transposon in the *RsMYB1-CACTA* promoter region may act as an enhancer promoting the expression of the gene.

Red-fleshed radish is a special variety in China, but its origin and evolution are unclear. Transposons have often functioned as modulators of gene expression during the domestication of crop species (Oikawa *et al.*, 2015). Furthermore, site-specific transposons can also be used to trace genetic relationships and origins (Zhang *et al.*, 2019). In this study, we determined that the CACTA transposon inserted upstream of the *RsMYB1* promoter is associated with the coloration of all red-fleshed accessions and some red-skinned varieties. A study on the inheritance of radish taproot skin and flesh color seemed to be relevant to the domestication and origin of red-fleshed radish. He *et al.* (1997) suggested that red-fleshed radish may have originated from the crossing of red-skinned and green-skinned radish plants. This report verified that the F_2_ population derived from a cross between red-skinned (white-fleshed) and green-skinned (white-fleshed) accessions included individual plants that produced a taproot with purple, red, green, or purple and green skin. A purple-skinned individual from the F_2_ population was self-crossed, producing a few red-fleshed and purple-skinned individuals. The self-crossing of these individuals generated progeny that were similar to red-fleshed radish. Therefore, we hypothesize that red-fleshed radish originated from a red-skinned radish containing the CACTA transposon. However, a thorough inheritance analysis involving diverse populations and genotyping is needed.

## Supplementary data

Supplementary data are available at *JXB* online.

Fig. S1. SNP-index graphs for the QTL-seq analysis.

Fig. S2. Insertion of the CACTA transposon leads to the spreading of DNA methylation in the JC01 mutant.

Fig. S3. *RsMYB1-CACTA* promoter (BS5 region) methylation level in the white-fleshed part and the red-fleshed part from chimeric MTH01 mutants.

Fig. S4. Phylogenetic tree of the *RsMYB1* and *RsMYB2* genes in the MTH01 and reference genomes.

Fig. S5. Anthocyanin biosynthesis pathway as well as the regulatory genes and their expression levels.

Table S1. Mutation rate of MTH01.

Table S2. Distribution of *RsMYB1-CACTA* in various cultivars.

Table S3. Details of the primers used in this study.

Table S4. Genome resequencing data.

Table S5. Details of all SNPs as well as the SNP-index and Δ(SNP-index).

Table S6. SNP-index and Δ(SNP-index) for each sliding window.

Table S7. All 16 candidate regions related to taproot flesh color according to the SNP-index based on the ‘Aokubi DH’ genome.

Table S8. Differentially expressed genes (MTH01/JC01) and annotations.

Table S9. Significantly enriched KEGG pathways associated with the DEGs.

Table S10. Expression levels of DEGs in candidate regions and annotations.

Table S11. Alignment of the *RsMYB1* and *RsMYB2* sequences in the MTH01 and reference genomes.

Table S12. Anthocyanin biosynthetic genes identified in *R. sativus* and their expression levels in MTH01 and JC01.

eraa010_suppl_Supplementary_file001

eraa010_suppl_Supplementary_file002

eraa010_suppl_Supplementary_file003

eraa010_suppl_Supplementary_file004

eraa010_suppl_Supplementary_file005

## Abbreviations:

5-azaC: 5-azacytidine
ABGs: anthocyanin biosynthetic genes
BS-seq: bisulfite sequencing
DEG: differentially expressed gene
QTL: quantitative trait locus
QTL-seq: quantitative trait location by genome resequencing
RPKM: reads per kilobase of million mapped reads
TRV: tobacco rattle virus
VIGS: virus-induced gene silencing

## References

[CIT0001] Ban Y, Honda C, Hatsuyama Y, Igarashi M, Bessho H, Moriguchi T 2007 Isolation and functional analysis of a MYB transcription factor gene that is a key regulator for the development of red coloration in apple skin. Plant & Cell Physiology 48, 958–970. 17526919 10.1093/pcp/pcm066

[CIT0002] Borevitz JO, Xia Y, Blount J, Dixon RA, Lamb C 2000 Activation tagging identifies a conserved MYB regulator of phenylpropanoid biosynthesis. The Plant Cell 12, 2383–2394. 11148285 10.1105/tpc.12.12.2383 PMC102225

[CIT0003] Butelli E, Titta L, Giorgio M, et al. 2008 Enrichment of tomato fruit with health-promoting anthocyanins by expression of select transcription factors. Nature Biotechnology 26, 1301–1308. 10.1038/nbt.1506 18953354

[CIT0004] BVRC, BAAFS (Beijing Vegetable Research Center, Beijing Academy of Agriculture and Forestry Science) 1977 Summary of the experiment of purification in “Xinlimei” radish. Beijing Agricultural Sciences 5, 50–56. (In Chinese)

[CIT0005] Cao X, Qiu Z, Wang X, et al. 2017 A putative *R3 MYB* repressor is the candidate gene underlying *atroviolacium*, a locus for anthocyanin pigmentation in tomato fruit. Journal of Experimental Botany 68, 5745–5758. 29186488 10.1093/jxb/erx382 PMC5854135

[CIT0006] Chiu LW, Zhou X, Burke S, Wu X, Prior RL, Li L 2010 The purple cauliflower arises from activation of a MYB transcription factor. Plant Physiology 154, 1470–1480. 20855520 10.1104/pp.110.164160 PMC2971621

[CIT0007] Cho MA, Min SR, Ko SM, Liu JR, Choi PS 2008 *Agrobacterium*-mediated genetic transformation of radish (*Raphanus sativus* L.). Plant Biotechnology 25, 205–208.

[CIT0008] Chopra S, Athma P, Li XG, Peterson T 1998 A maize *Myb* homolog is encoded by a multicopy gene complex. Molecular & General Genetics 260, 372–380. 9870702 10.1007/s004380050906

[CIT0009] Espley RV, Brendolise C, Chagné D, et al. 2009 Multiple repeats of a promoter segment causes transcription factor autoregulation in red apples. The Plant Cell 21, 168–183. 19151225 10.1105/tpc.108.059329 PMC2648084

[CIT0010] Falcone Ferreyra ML, Rius S, Emiliani J, Pourcel L, Feller A, Morohashi K, Casati P, Grotewold E 2010 Cloning and characterization of a UV-B-inducible maize flavonol synthase. The Plant Journal 62, 77–91. 20059741 10.1111/j.1365-313X.2010.04133.x

[CIT0011] Furukawa T, Maekawa M, Oki T, Suda I, Iida S, Shimada H, Takamure I, Kadowaki K 2007 The *Rc* and *Rd* genes are involved in proanthocyanidin synthesis in rice pericarp. The Plant Journal 49, 91–102. 17163879 10.1111/j.1365-313X.2006.02958.x

[CIT0012] Gonzalez A, Zhao M, Leavitt JM, Lloyd AM 2008 Regulation of the anthocyanin biosynthetic pathway by the *TTG1/bHLH/Myb* transcriptional complex in *Arabidopsis* seedlings. The Plant Journal 53, 814–827. 18036197 10.1111/j.1365-313X.2007.03373.x

[CIT0013] Grotewold E, Drummond BJ, Bowen B, Peterson T 1994 The *myb*-homologous *P* gene controls phlobaphene pigmentation in maize floral organs by directly activating a flavonoid biosynthetic gene subset. Cell 76, 543–553. 8313474 10.1016/0092-8674(94)90117-1

[CIT0014] Guo N, Cheng F, Wu J, Liu B, Zheng S, Liang J, Wang X 2014 Anthocyanin biosynthetic genes in *Brassica rapa*. BMC Genomics 15, 426. 24893600 10.1186/1471-2164-15-426 PMC4072887

[CIT0015] Harborne JB, Paxman GJ 1964 Genetics of anthocyanin product in the radish. Heredity 19, 505–506. 14202705 10.1038/hdy.1964.57

[CIT0016] He J, Giusti MM 2010 Anthocyanins: natural colorants with health-promoting properties. Annual Review of Food Science and Technology 1, 163–187. 10.1146/annurev.food.080708.100754 22129334

[CIT0017] He QW, Zhao SY, Shi HL, An ZQ, Lang FQ, Wang SF 1997 Preliminary study on skin color inheritance of Chinese radish. Shandong Agricultural Sciences 2, 4–9. (In Chinese)

[CIT0018] Kobayashi S, Goto-Yamamoto N, Hirochika H 2004 Retrotransposon-induced mutations in grape skin color. Science 304, 982. 15143274 10.1126/science.1095011

[CIT0019] Koes R, Verweij W, Quattrocchio F 2005 Flavonoids: a colorful model for the regulation and evolution of biochemical pathways. Trends in Plant Science 10, 236–242. 15882656 10.1016/j.tplants.2005.03.002

[CIT0020] Langmead B, Trapnell C, Pop M, Salzberg SL 2009 Ultrafast and memory-efficient alignment of short DNA sequences to the human genome. Genome Biology 10, R25. 19261174 10.1186/gb-2009-10-3-r25 PMC2690996

[CIT0021] Li H, Durbin R 2009 Fast and accurate short read alignment with Burrows-Wheeler transform. Bioinformatics 25, 1754–1760. 19451168 10.1093/bioinformatics/btp324 PMC2705234

[CIT0022] Lim SH, Kim DH, Kim JK, Lee JY, Ha SH 2017 A radish basic helix-loop-helix transcription factor, *RsTT8* acts a positive regulator for anthocyanin biosynthesis. Frontiers in Plant Science 8, 1917. 29167678 10.3389/fpls.2017.01917 PMC5682339

[CIT0023] Lim SH, Song JH, Kim DH, Kim JK, Lee JY, Kim YM, Ha SH 2016 Activation of anthocyanin biosynthesis by expression of the radish R2R3-MYB transcription factor gene *RsMYB1*. Plant Cell Reports 35, 641–653. 26703384 10.1007/s00299-015-1909-3

[CIT0024] Lin LZ, Harnly JM 2007 A screening method for the identification of glycosylated flavonoids and other phenolic compounds using a standard analytical approach for all plant materials. Journal of Agricultural and Food Chemistry 55, 1084–1096. 17256956 10.1021/jf062431s PMC3762687

[CIT0025] Lister R, O’Malley RC, Tonti-Filippini J, Gregory BD, Berry CC, Millar AH, Ecker JR 2008 Highly integrated single-base resolution maps of the epigenome in *Arabidopsis*. Cell 133, 523–536. 18423832 10.1016/j.cell.2008.03.029 PMC2723732

[CIT0026] Liu Y, Schiff M, Dinesh-Kumar SP 2002 Virus-induced gene silencing in tomato. The Plant Journal 31, 777–786. 12220268 10.1046/j.1365-313x.2002.01394.x

[CIT0027] Liu TJ, Zhang YJ, Zhang XH, Sun YY, Wang HP, Song JP, Li XX, 2019 Transcriptome analyses reveal key genes involved in skin color changes of ‘Xinlimei’ radish taproot. Plant Physiology and Biochemistry 139, 528–539. 31029026 10.1016/j.plaphy.2019.04.006

[CIT0028] Luo XB, Xu L, Wang Y, Dong JH, Chen YL, Tang MJ, Fan LX, Zhu YL, Liu LW 2020 An ultra-high density genetic map provides insights into genome synteny, recombination landscape and taproot skin color in radish (*Raphanus sativus* L.). Plant Biotechnology Journal 18, 274–286. 31218798 10.1111/pbi.13195 PMC6920339

[CIT0029] Martin A, Troadec C, Boualem A, Rajab M, Fernandez R, Morin H, Pitrat M, Dogimont C, Bendahmane A 2009 A transposon-induced epigenetic change leads to sex determination in melon. Nature 461, 1135–1138. 19847267 10.1038/nature08498

[CIT0030] Masukawa T, Cheon KS, Mizuta D, Nakatsuka A, Kobayashi N 2018 Insertion of a retrotransposon into a *flavonoid 3′-hydroxylase* homolog confers the red root character in the radish (*Raphanus sativus* L. var. *longipinnatus* L. H. Bailey). The Horticulture Journal 87, 89–96.

[CIT0031] McKenna A, Hanna M, Banks E, et al. 2010 The genome analysis toolkit: a MapReduce framework for analyzing next-generation DNA sequencing data. Genome Research 20, 1297–1303. 20644199 10.1101/gr.107524.110 PMC2928508

[CIT0032] Mitsui Y, Shimomura M, Komatsu K, et al. 2015 The radish genome and comprehensive gene expression profile of tuberous root formation and development. Scientific Reports 5, 10835. 26056784 10.1038/srep10835 PMC4650646

[CIT0033] Muleke EM, Fan L, Wang Y, Xu L, Zhu X, Zhang W, Cao Y, Karanja BK, Liu L 2017 Coordinated regulation of anthocyanin biosynthesis genes confers varied phenotypic and spatial-temporal anthocyanin accumulation in radish (*Raphanus sativus* L.). Frontiers in Plant Science 8, 1243. 28769952 10.3389/fpls.2017.01243 PMC5515825

[CIT0034] Murray MG, Thompson WF 1980 Rapid isolation of high molecular weight plant DNA. Nucleic Acids Research 8, 4321–4325. 7433111 10.1093/nar/8.19.4321 PMC324241

[CIT0035] Oikawa T, Maeda H, Oguchi T, Yamaguchi T, Tanabe N, Ebana K, Yano M, Ebitani T, Izawa T 2015 The birth of a black rice gene and its local spread by introgression. The Plant Cell 27, 2401–2414. 26362607 10.1105/tpc.15.00310 PMC4815089

[CIT0036] Park M, Lee JH, Han K, Jang S, Han J, Lim JH, Jung JW, Kang BC 2019 A major QTL and candidate genes for capsaicinoid biosynthesis in the pericarp of *Capsicum chinense* revealed using QTL-seq and RNA-seq. Theoretical and Applied Genetics 132, 515–529. 30426173 10.1007/s00122-018-3238-8

[CIT0037] Park NI, Xu H, Li X, Jang IH, Park S, Ahn GH, Lim YP, Kim SJ, Park SU 2011 Anthocyanin accumulation and expression of anthocyanin biosynthetic genes in radish (*Raphanus sativus*). Journal of Agricultural and Food Chemistry 59, 6034–6039. 21548630 10.1021/jf200824c

[CIT0038] Petroni K, Tonelli C 2011 Recent advances on the regulation of anthocyanin synthesis in reproductive organs. Plant Science 181, 219–229. 21763532 10.1016/j.plantsci.2011.05.009

[CIT0039] Saxena RK, Penmetsa RV, Upadhyaya HD, et al. 2012 Large-scale development of cost-effective single-nucleotide polymorphism marker assays for genetic mapping in pigeonpea and comparative mapping in legumes. DNA Research 19, 449–461. 23103470 10.1093/dnares/dss025 PMC3514856

[CIT0040] Shi MZ, Xie DY 2014 Biosynthesis and metabolic engineering of anthocyanins in *Arabidopsis thaliana*. Recent Patents on Biotechnology 8, 47–60. 24354533 10.2174/1872208307666131218123538 PMC4036305

[CIT0041] Sidorenko LV, Li X, Cocciolone SM, Chopra S, Tagliani L, Bowen B, Daniels M, Peterson T 2000 Complex structure of a maize *Myb* gene promoter: functional analysis in transgenic plants. The Plant Journal 22, 471–482. 10886767 10.1046/j.1365-313x.2000.00750.x

[CIT0042] Solís MT, El-Tantawy AA, Cano V, Risueño MC, Testillano PS 2015 5-azacytidine promotes microspore embryogenesis initiation by decreasing global DNA methylation, but prevents subsequent embryo development in rapeseed and barley. Frontiers in Plant Science 6, 472. 26161085 10.3389/fpls.2015.00472 PMC4479788

[CIT0043] Studer A, Zhao Q, Ross-Ibarra J, Doebley J 2011 Identification of a functional transposon insertion in the maize domestication gene *tb1*. Nature Genetics 43, 1160–1163. 21946354 10.1038/ng.942 PMC3686474

[CIT0044] Sun Y, Qiu Y, Duan M, Wang J, Zhang X, Wang H, Song J, Li X 2017 Identification of anthocyanin biosynthesis related microRNAs in a distinctive Chinese radish (*Raphanus sativus* L.) by high-throughput sequencing. Molecular Genetics and Genomics 292, 215–229. 27817120 10.1007/s00438-016-1268-y

[CIT0045] Sun Y, Wang J, Qiu Y, Liu T, Song J, Li X 2018 Identification of ‘Xinlimei’ radish candidate genes associated with anthocyanin biosynthesis based on a transcriptome analysis. Gene 657, 81–91. 29518548 10.1016/j.gene.2018.03.001

[CIT0046] Sweeney MT, Thomson MJ, Pfeil BE, McCouch S 2006 Caught red-handed: *Rc* encodes a basic helix-loop-helix protein conditioning red pericarp in rice. The Plant Cell 18, 283–294. 16399804 10.1105/tpc.105.038430 PMC1356539

[CIT0047] Takagi H, Abe A, Yoshida K, et al. 2013 QTL-seq: rapid mapping of quantitative trait loci in rice by whole genome resequencing of DNA from two bulked populations. The Plant Journal 74, 174–183. 23289725 10.1111/tpj.12105

[CIT0048] Tang K, Lang Z, Zhang H, Zhu JK 2016 The DNA demethylase ROS1 targets genomic regions with distinct chromatin modifications. Nature Plants 2, 16169. 27797352 10.1038/nplants.2016.169 PMC5123759

[CIT0049] Tatebe T 1938 On inheritance of root color in *Raphanus sativus* L. Japanese Journal of Genetics 14, 39–50.

[CIT0050] Telias A, Lin-Wang K, Stevenson DE, Cooney JM, Hellens RP, Allan AC, Hoover EE, Bradeen JM 2011 Apple skin patterning is associated with differential expression of *MYB10*. BMC Plant Biology 11, 93. 21599973 10.1186/1471-2229-11-93 PMC3127826

[CIT0051] Tian PF 2006 Progress in plant CACTA elements. Acta Genetica Sinica 33, 765–774. 16980122 10.1016/S0379-4172(06)60109-1

[CIT0052] Tohge T, Nishiyama Y, Hirai MY, et al. 2005 Functional genomics by integrated analysis of metabolome and transcriptome of Arabidopsis plants over-expressing an MYB transcription factor. The Plant Journal 42, 218–235. 15807784 10.1111/j.1365-313X.2005.02371.x

[CIT0053] Trapnell C, Pachter L, Salzberg SL 2009 TopHat: discovering splice junctions with RNA-Seq. Bioinformatics 25, 1105–1111. 19289445 10.1093/bioinformatics/btp120 PMC2672628

[CIT0054] Tyč D, Nocarová E, Sikorová L, Fischer L 2017 5-Azacytidine mediated reactivation of silenced transgenes in potato (*Solanum tuberosum*) at the whole plant level. Plant Cell Reports 36, 1311–1322. 28510781 10.1007/s00299-017-2155-7

[CIT0055] Van Ooijen JW 2006 JoinMap® 4, software for the calculation of genetic linkage maps in experimental populations. Wageningen: Kyazma BV.

[CIT0056] Wagner GP, Kin K, Lynch VJ 2012 Measurement of mRNA abundance using RNA-seq data: RPKM measure is inconsistent among samples. Theory in Biosciences 131, 281–285. 22872506 10.1007/s12064-012-0162-3

[CIT0057] Walker AR, Lee E, Bogs J, McDavid DA, Thomas MR, Robinson SP 2007 White grapes arose through the mutation of two similar and adjacent regulatory genes. The Plant Journal 49, 772–785. 17316172 10.1111/j.1365-313X.2006.02997.x

[CIT0058] Wang L, Feng Z, Wang X, Wang X, Zhang X 2010 DEGseq: an R package for identifying differentially expressed genes from RNA-seq data. Bioinformatics 26, 136–138. 19855105 10.1093/bioinformatics/btp612

[CIT0059] Wang Z, Meng D, Wang A, Li T, Jiang S, Cong P, Li T 2013 The methylation of the *PcMYB10* promoter is associated with green-skinned sport in Max Red Bartlett pear. Plant Physiology 162, 885–896. 23629835 10.1104/pp.113.214700 PMC3668077

[CIT0060] Wittmeyer K, Cui J, Chatterjee D, et al. 2018 The dominant and poorly penetrant phenotypes of maize *unstable factor for orange1* are caused by DNA methylation changes at a linked transposon. The Plant Cell 30, 3006–3023. 30563848 10.1105/tpc.18.00546 PMC6354275

[CIT0061] Yi G, Kim JS, Park JE, Shin H, Yu SH, Park S, Huh JH 2018 *MYB1* transcription factor is a candidate responsible for red root skin in radish (*Raphanus sativus* L.). PLOS ONE 13, e0204241. 30240413 10.1371/journal.pone.0204241 PMC6150496

[CIT0062] Zhang L, Hu J, Han X, et al. 2019 A high-quality apple genome assembly reveals the association of a retrotransposon and red fruit colour. Nature Communications 10, 1494. 10.1038/s41467-019-09518-x PMC6445120 30940818

[CIT0063] Zhang H, Lang Z, Zhu JK 2018 Dynamics and function of DNA methylation in plants. Nature Reviews. Molecular Cell Biology 19, 489–506. 29784956 10.1038/s41580-018-0016-z

[CIT0064] Zhang X, Yazaki J, Sundaresan A, et al. 2006 Genome-wide high-resolution mapping and functional analysis of DNA methylation in *Arabidopsis*. Cell 126, 1189–1201. 16949657 10.1016/j.cell.2006.08.003

[CIT0065] Zhang X, Yue Z, Mei S, et al. 2015 A *de novo* genome of a Chinese radish cultivar. Horticultural Plant Journal 1, 155–164.

[CIT0066] Zhu J, Fang L, Yu J, Zhao Y, Chen F, Xia G 2018 5-Azacytidine treatment and *TaPBF-D* over-expression increases glutenin accumulation within the wheat grain by hypomethylating the *Glu-1* promoters. Theoretical and Applied Genetics 131, 735–746. 29214328 10.1007/s00122-017-3032-z

