# Supplementary material for: Transposon-induced methylation of the RsMYB1 promoter disturbs anthocyanin accumulation in red-fleshed radish
Source: J Exp Bot. 2020 Jan 21;71(9):2537–50. doi: 10.1093/jxb/eraa010 (PMC7210773; doi:10.1093/jxb/eraa010)
Supplement: eraa010_suppl_Supplementary_file005 [file eraa010_suppl_supplementary_file005.pdf]

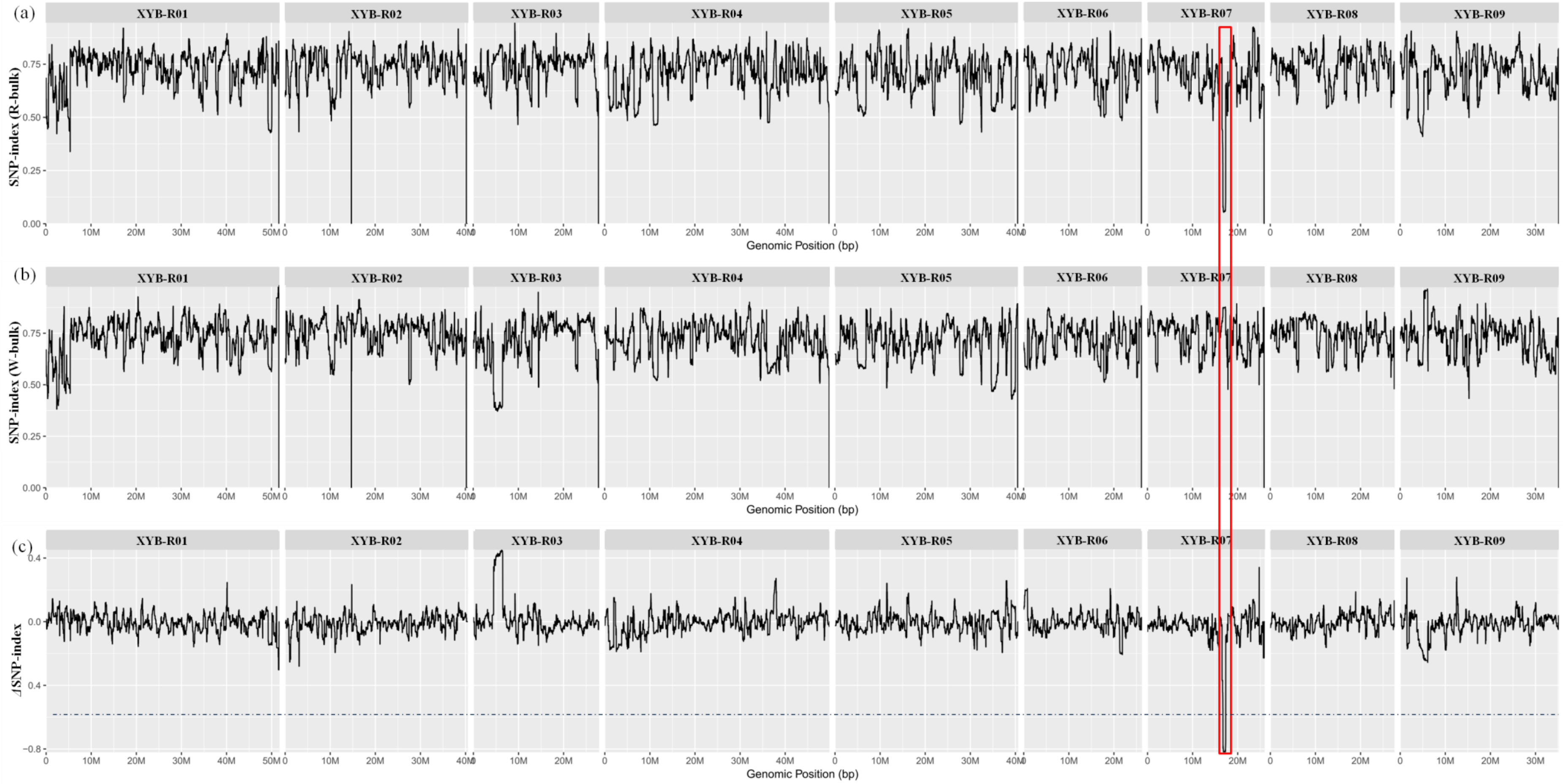

Figure S1. SNP-index graphs for the red-fleshed bulk (a) and the white-fleshed bulk (b) as well as the Δ(SNP-index) graph (c) for the QTL-seq analysis. The *x-axis* represents the positions of nine chromosomes and the *y-axis* represents the SNP-index. A candidate QTL was identified on chromosome 7 (16.25–18.33 Mb interval) with the criterion that the Δ(SNP-index) was greater than 0.7 ($P < 0.05$).

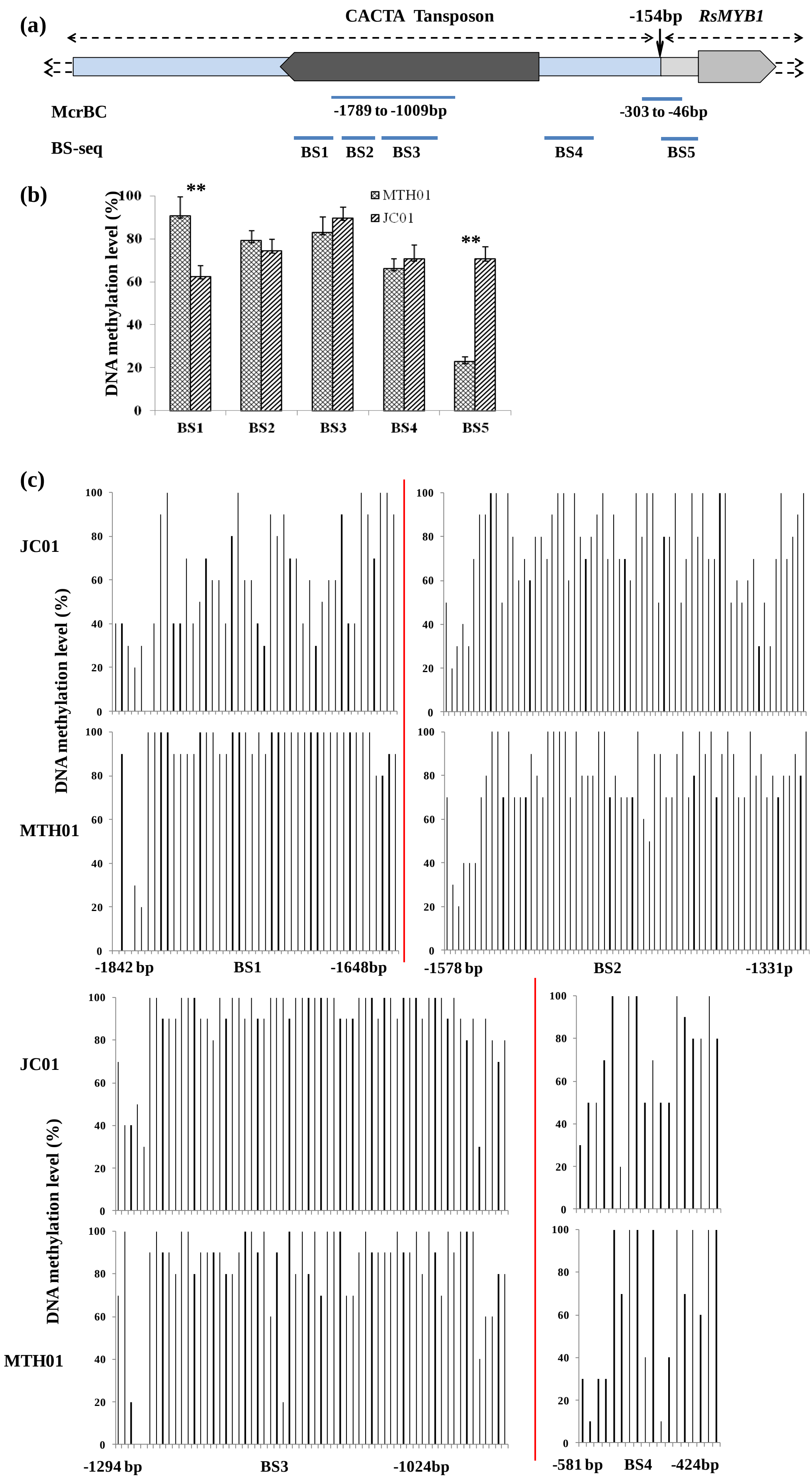

(a)
CACTA Tansposon
-154bp
RsMYB1
McrBC
-1789 to -1009bp
-303 to -46bp
BS-seq
BS1
BS2
BS3
BS4
BS5
(b)
DNA methylation level (%)
MTH01
JC01
**
**
BS1
BS2
BS3
BS4
BS5
(c)
JC01
MTH01
DNA methylation level (%)
-1842 bp
BS1
-1648bp
-1578 bp
BS2
-1331p
-1294 bp
BS3
-1024bp
-581 bp
BS4
-424bp

Figure S2. Insertion of the CACTA transposon leads to the spreading of DNA methylation in the JC01 mutant. (a) Structural representation and location of the CACTA transposon inserted upstream of *RsMYB1*. The DNA methylation of the BS1–BS5 regions was analyzed by BS-seq. (b) Quantitative analysis of the methylation in the region upstream of *RsMYB1-CACTA* in MTH01 and JC01 by BS-seq. The bars represent the average of all cytosine methylation percentages in this region. ** means significantly different (P = 0.05). Data are presented as the mean and standard deviation. (c) Analysis of the cytosine methylation of the *RsMYB1-CACTA* promoter region by BS-seq. The following locations indicated in (a) were examined: BS1 (−1,842 bp to −1,648 bp), BS2 (−1,578 bp to −1,331 bp), BS3 (−1,294 bp to −1,024 bp) and BS4 (−581 bp to −424bp) relative to the *RsMYB1-CACTA* start codon (ATG). The percentage of each methylated cytosine in this region is indicated by vertical bars.

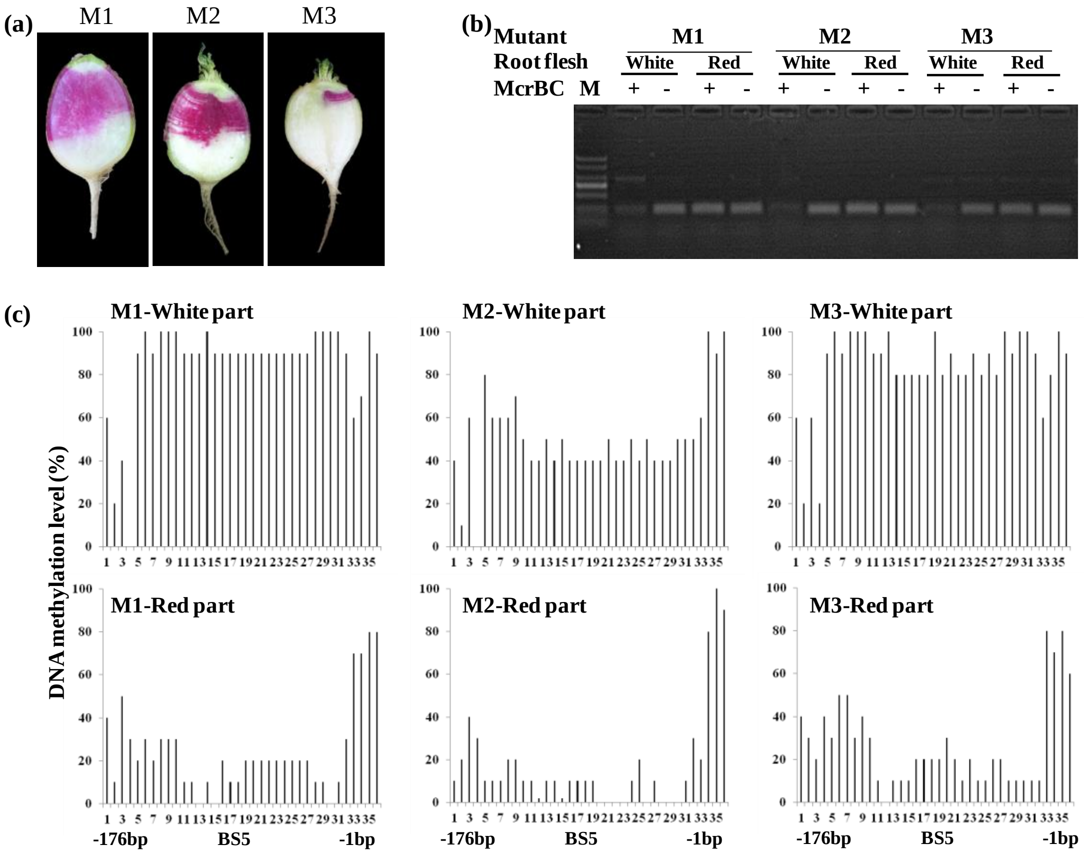

Figure S3. The *RsMYB1-CACTA* promoter (BS5 region) was more highly methylated in the DNA extracted from the white-fleshed part than in the DNA extracted from the red-fleshed part of chimeric MTH01 mutants. (a) Phenotype of chimeric MTH01 mutants. (b) McrBC-sensitive PCR analysis of *RsMYB1-CACTA* promoter regions (−46 bp to −303 bp) in the red-fleshed and white-fleshed parts of mutants M1, M2, and M3. + and − indicate whether genomic DNA was treated with McrBC before the PCR amplification. The absence of a PCR product for the McrBC-treated samples indicates the DNA was methylated. (c) Analysis of the cytosine methylation of the promoter [BS5 region (−176 bp to −1 bp relative to the *RsMYB1-CACTA* ATG)] in the red-fleshed and white-fleshed parts of mutants M1, M2, and M3 by BS-seq. The percentage of each methylated cytosine in the region is indicated by vertical bars.

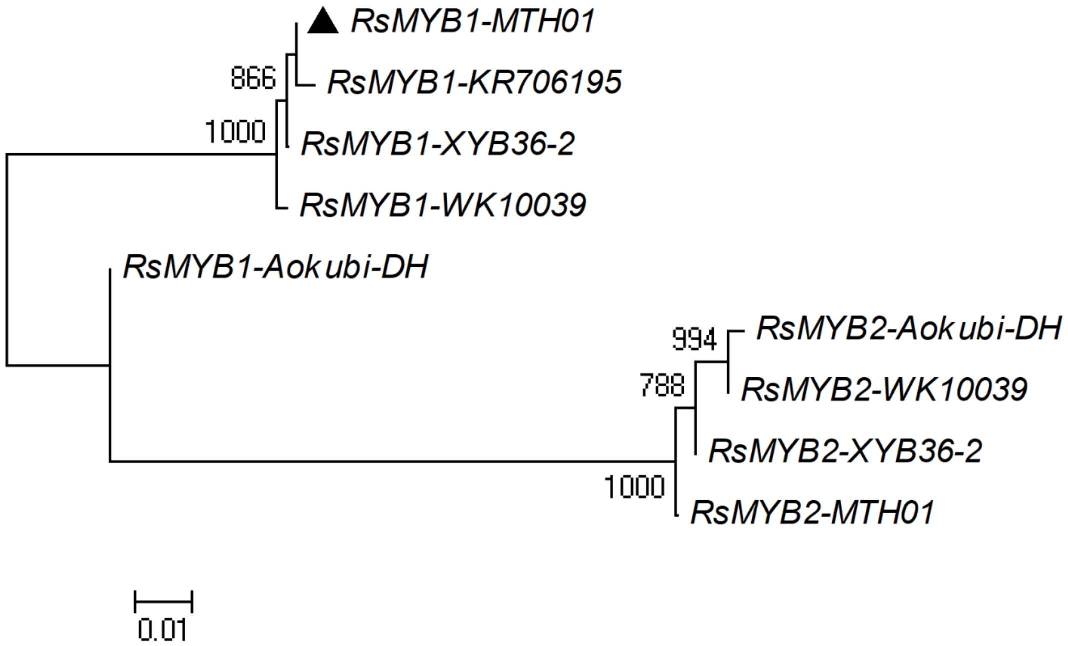

Figure S4. Phylogenetic tree of the *RsMYB1* and *RsMYB2* genes in the MTH01 and reference genomes. The following sequences (*RsMYB1* and *RsMYB2,* respectively) from the reference genomes are included in the phylogenetic tree: 'Aokubi DH': RSG33469 and RSG19108; 'WK10039': Rs384850 and Rs388430; and 'XYB36-2': Rsa10033919 and Rsa10034073. The numbers at branch nodes represent bootstrap values after 1,000 replicates. The scale bars indicate nucleotide substitutions per site.

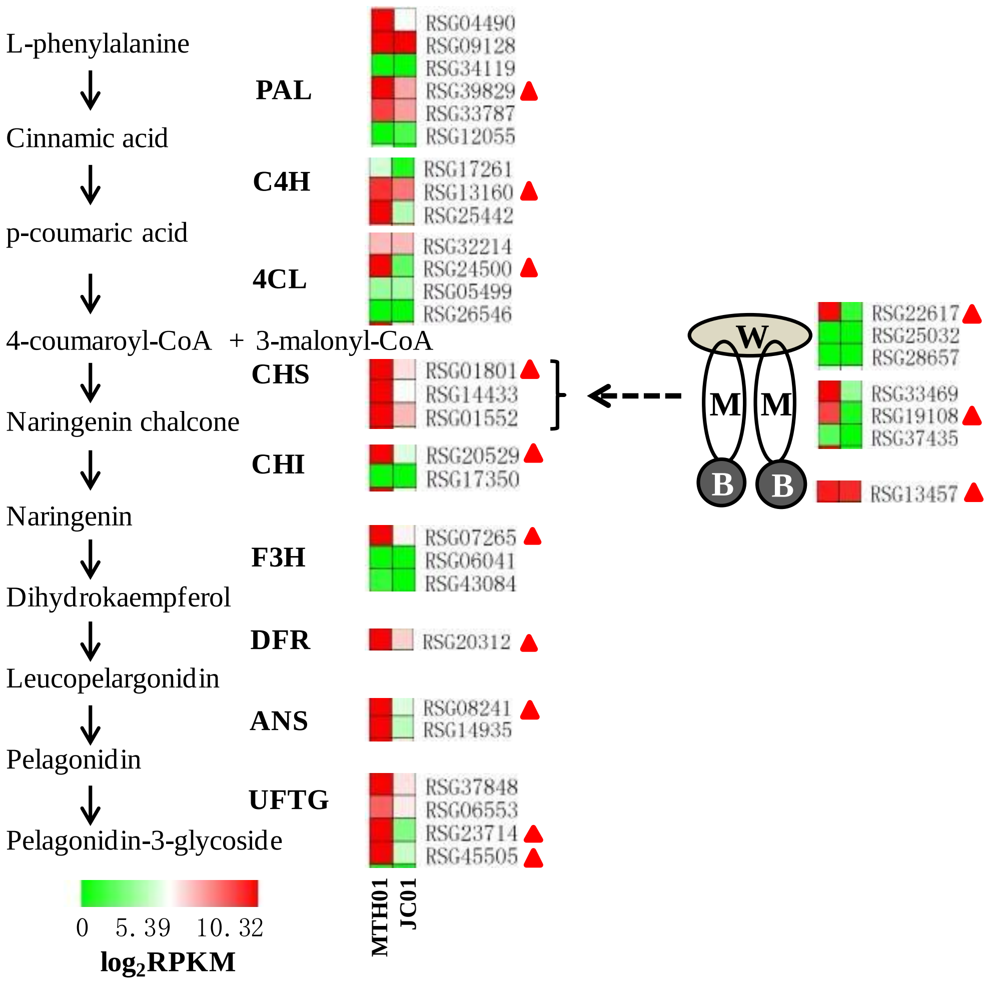

Figure S5. Anthocyanin biosynthesis pathway as well as the regulatory genes and their expression levels. The heatmap presents the expression levels of anthocyanin biosynthetic genes based on RNA-seq data. Expression data represent the log_2 RPKM of the root flesh from MTH01 and JC01 samples. A red triangle indicates the gene has been cloned in radish. PAL, phenylalanine ammonia lyase; C4H, cinnamic acid 4-hydroxylase; 4CL, 4-coumarate CoA ligase; CHS, chalcone synthase; CHI, chalcone isomerase; F3H, flavanone-3-hydroxylase; DFR, dihydroflavonol reductase; ANS, anthocyanidin synthase; UFGT, UDP-glucose:flavonoid-3-O-glucosyltransferase; bHLH, basic helix–loop–helix; and WD40, WD-repeat protein. The heatmap was prepared with the Cluster 3.0 program.
